# High spatial resolution global ocean metagenomes from Bio-GO-SHIP repeat hydrography transects

**DOI:** 10.1038/s41597-021-00889-9

**Published:** 2021-04-16

**Authors:** Alyse A. Larkin, Catherine A. Garcia, Nathan Garcia, Melissa L. Brock, Jenna A. Lee, Lucas J. Ustick, Leticia Barbero, Brendan R. Carter, Rolf E. Sonnerup, Lynne D. Talley, Glen A. Tarran, Denis L. Volkov, Adam C. Martiny

**Affiliations:** 1 Department of Earth System Science, University of California at Irvine, Irvine, CA USA; 2 Department of Ecology and Evolutionary Biology, University of California at Irvine, Irvine, CA USA; 3 NOAA Atlantic Oceanographic and Meteorological Laboratory, Miami, FL USA; 4 Cooperative Institute for Marine & Atmospheric Studies, University of Miami, Miami, FL USA; 5 NOAA Pacific Marine Environmental Laboratory, Seattle, WA USA; 6 Cooperative Institute for Climate, Ocean, and Ecosystem Studies, University of Washington, Seattle, WA USA; 7 Climate, Atmospheric Sciences, and Physical Oceanography Division, Scripps Institution of Oceanography, University of California, San Diego, CA USA; 8 Plymouth Marine Laboratory, Plymouth, UK

**Keywords:** Element cycles, Marine biology, Metagenomics

## Abstract

Detailed descriptions of microbial communities have lagged far behind physical and chemical measurements in the marine environment. Here, we present 971 globally distributed surface ocean metagenomes collected at high spatio-temporal resolution. Our low-cost metagenomic sequencing protocol produced 3.65 terabases of data, where the median number of base pairs per sample was 3.41 billion. The median distance between sampling stations was 26 km. The metagenomic libraries described here were collected as a part of a biological initiative for the Global Ocean Ship-based Hydrographic Investigations Program, or “Bio-GO-SHIP.” One of the primary aims of GO-SHIP is to produce high spatial and vertical resolution measurements of key state variables to directly quantify climate change impacts on ocean environments. By similarly collecting marine metagenomes at high spatiotemporal resolution, we expect that this dataset will help answer questions about the link between microbial communities and biogeochemical fluxes in a changing ocean.

## Abstract (summary)

**Table:** 

Measurement(s)	DNA sequencing • temperature of water • concentration of phosphate in water • concentration of nitrogen atom in water
Technology Type(s)	Illumina sequencing • watercraft • continuous flow autoanalyzer
Sample Characteristic - Organism	marine metagenome
Sample Characteristic - Environment	ocean
Sample Characteristic - Location	global

Machine-accessible metadata file describing the reported data: 10.6084/m9.figshare.13971122

## Background & Summary

A growing list of coordinated scientific efforts have produced deep metagenomic libraries of the surface ocean. Projects such as the Global Ocean Survey, Tara Oceans, and bioGEOTRACES^1–3^ have significantly advanced our understanding of marine microbial biogeography and biodiversity. However, this ever-increasing abundance of metagenomic data raises the question of how do we move beyond analyses of biodiversity to linking microbial traits with ecosystem function and elemental fluxes^4^. In oceanography, it has been widely acknowledged that sparse sampling results in high noise and error rates that in turn prevent the characterization of dynamic chemical balances and limit biogeochemical models^5^. Thus, we propose that an increased emphasis on high resolution spatio-temporal sampling of marine microbial communities would allow for a more mechanistic understanding of the relationship between microbes and ocean biogeochemistry.

The Global Ocean Ship-based Hydrographic Investigations Program (GO-SHIP) seeks to produce high spatial and vertical resolution measurements of physical, chemical, and biological parameters over the full water column. This internationally-organized program coordinates a network of sustained hydrographic sections that are repeatedly measured on an approximately decadal time scale. Compared to autonomous programs such as Argo, which has significantly increased the spatial and temporal resolution of ocean observations^6^, ship-based programs have the advantage of a much broader range of biogeochemical measurement capabilities and full water column coverage. To date, repeat hydrography programs have largely focused on physical (light, currents, water column thermohaline structure, etc.) and chemical (nutrients, oxygen, dissolved organic and inorganic carbon, pH, etc.) state variables. This work has significantly improved our understanding of the response of oxygen^7^, pH^8^, calcium carbonate saturation depth^9^, and sea level rise^10^ to global warming and anthropogenic carbon accumulation^11^. By comparison, systematic and sustained biological measurements of the microbial component of ocean ecosystems has lagged far behind.

**Fig. 1:**
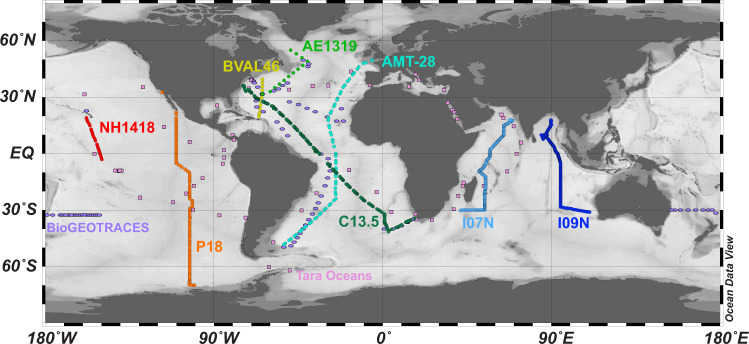
Distribution of global surface microbial metagenomes from Bio-GO-SHIP (circles) in comparison to Tara Oceans (squares) and bioGEOTRACES (ovals). Symbol colours match the corresponding cruise name label colour.

**Fig. 2:**
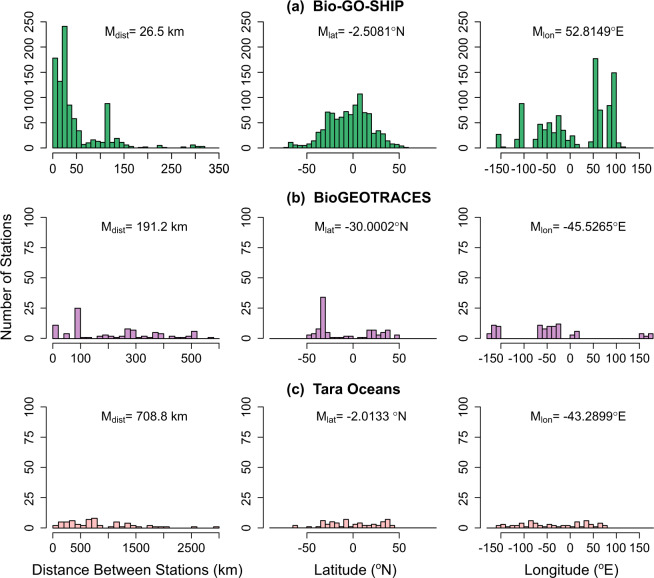
Comparison of the distance between stations, station latitudes, and station longitudes for global surface ocean metagenomes. Individual station locations from (**A**) Bio-GO-SHIP, (**B**) bioGEOTRACES and (**C**) Tara Oceans were examined. Plots are labelled with the median value, M. Station distance was calculated as the distance to the nearest station.

We present a dataset of 971 ocean surface water metagenomes collected at high spatio-temporal resolution in an effort to more mechanistically link marine microbial traits and biodiversity to both chemical and hydrodynamic ecosystem fluxes as a part of a novel Bio-GO-SHIP sampling program. Samples were collected in the Atlantic, Pacific, and Indian Ocean basins (Fig. 1, Table 1). This effort has been supported by GO-SHIP, SOCCOM, the Plymouth Marine Laboratory Atlantic Meridional Transect (PML AMT), and three National Science Foundation (NSF) Dimensions of Biodiversity funded cruises (AE1319, BVAL46, and NH1418) (Table 2). Whereas the median distance between Tara Oceans sampling stations was 709 km and the median distance between bioGEOTRACES sampling stations was 191 km, the median distance between sampling stations in the current Bio-GO-SHIP dataset is 26.5 km (Fig. 2). In addition, the majority of Bio-GO-SHIP samples were collected every 4–6 hours, allowing for analysis of diel fluctuations in microbial composition and gene content^12^. We anticipate that our high-resolution sampling scheme will allow for a more detailed examination of the relationship between the broad range of geochemical parameters measured across the various cruises (Table 2) and microbial diversity and traits.

**Table 1:** Sampling protocols and read counts for global Bio-GO-SHIP surface ocean metagenomes.

Cruise/Year	DNA Collection	DNA Volume	Station Count	Total Reads	Total Bases	Median Bases Per Sample	Range of Bases Per Sample	Total Pre-Filter Reads ≥ Q25
I07N 2018	Underway, CTD	4 L, 2-4 L	248	6.20 × 10^9^	9.36 × 10^11^	3.27 × 10^9^	2.47 × 10^8^–1.42 × 10^10^	4.65 × 10^9^
I09N 2016	Underway	10 L	242	5.73 × 10^9^	8.64 × 10^11^	3.10 × 10^9^	4.71 × 10^8^–1.22 × 10^10^	4.15 × 10^9^
C13.5 2020	Underway	5-10 L	229	5.94 × 10^9^	8.96 × 10^11^	2.94 × 10^9^	3.98 × 10^8^–2.14 × 10^10^	4.17 × 10^9^
P18 2016-17	CTD	2 L	104	3.22 × 10^9^	4.86 × 10^11^	4.46 × 10^9^	6.14 × 10^7^–1.77 × 10^10^	2.55 × 10^9^
AMT-28 2018	CTD	2 L	63	2.18 × 10^9^	3.29 × 10^11^	4.95 × 10^9^	1.62 × 10^9^–1.22 × 10^10^	1.71 × 10^9^
NH1418 2014	CTD	2 L	23	5.41 × 10^8^	8.17 × 10^10^	3.03 × 10^9^	2.42 × 10^9^–1.08 × 10^10^	4.02 × 10^8^
AE1319 2013	CTD	2 L	13	2.01 × 10^8^	3.03 × 10^10^	4.69 × 10^9^	2.15 × 10^9^–7.43 × 10^9^	1.63 × 10^8^
BVAL46 2011	CTD	2 L	12	2.01 × 10^8^	3.04 × 10^10^	2.73 × 10^9^	2.33 × 10^9^–4.88 × 10^9^	1.61 × 10^8^

**Table 2:** Publicly available metadata variables collected on Bio-GO-SHIP cruises.

Campaign	Data Host and License	DOI	Metadata Variables
I07N, GO-SHIP	CCDHO; PDM	10.7942/C25H2B	Temperature, Salinity, Dissolved O_2_, Nutrients (NO_3_, NO_2_, PO_4_, SiO_4_), Chlorofluorocarbons (CFCs) /SF_6_, Dissolved Inorganic Carbon, Dissolved Organic Carbon, Total pH, Total Alkalinity, Stable gases (N_2_O), Calcium
I09N, GO-SHIP	CCDHO; PDM	10.7942/C2008W	Temperature, Salinity, Dissolved O_2_, Nutrients (NO_3_, NO_2_, NH_4_, PO_4_, SiO_4_), Chlorofluorocarbons (CFCs) /SF_6_, ^13^C and ^14^C of DIC, Dissolved Inorganic Carbon, Dissolved Organic Carbon, Total pH, Total Alkalinity, Stable gases (N_2_, N_2_O, Ar), ^18^O, Chromophoric Dissolved Organic Matter (CDOM), Pigment HPLC, Chlorophyll A, Dissolved/ particulate/ cellular P and Fe, N P and Fe uptake rates
C13.5*/A13.5, GO-SHIP	CCDHO; PDM	10.7942/C2894Zs	Temperature, Salinity, Dissolved O_2_, Chlorophyll fluorometer and scattering
P18, GO-SHIP	CCDHO; PDM	10.7942/C21T0F	Temperature, Salinity, Dissolved O_2_, Nutrients (NO_3_, NO_2_, PO_4_, SiO_4_), Chlorofluorocarbons (CFCs) /SF_6_, Dissolved Inorganic Carbon (DIC), Total pH, Total Alkalinity
AMT-28, PML AMT SOCCOM, NSF	BODC, NERC Open Government License SOCCOM, PDM	https://doi.org/10/fqkd	Temperature, Salinity, Dissolved O_2_, Density, Fluorescence, PAR Irradiance, *et al*.
NH1418, NSF	BCO-DMO, WHOAS; CC BY 4.0	10.26008/1912/bco-dmo.829895.1	Temperature, Salinity, Dissolved O_2_ / Saturation, Density, Chlorophyll a, PAR irradiance, Fluorescence, Nutrients (NO_3_ + NO_2_, NO_2_), Soluble Reactive Phosphorus (SRP), Particulate Organic C N and P, *Prochlorococcus*/ *Synechococcus*/ Picoeukaryote/ Nanoeukaryote / Croccosphera cell counts and POC/cell
AE1319, NSF	BCO-DMO, WHOAS; CC BY 4.0	10.26008/1912/bco-dmo.829797.1 10.26008/1912/bco-dmo.538091.2	Temperature, Salinity, Dissolved O_2_, PAR irradiance, Chlorophyll a, Nutrients (NO_3_ + NO_2_, PO_4_, SiO_4_), Soluble Reactive Phosphorus (SRP), Particulate Organic C N and P, *Prochlorococcus*/ *Synechococcus*/ Picoeukaryote/ Nanoeukaryote cell counts
BVAL46, NSF, BATS	BCO-DMO, WHOAS; CC BY 4.0	10.26008/1912/bco-dmo.829843.1 10.26008/1912/bco-dmo.538091.2	Temperature, Salinity, Dissolved O_2_, Chlorophyll a, Nutrients (NO_3_ + NO_2_, PO_4_, SiO_4_), Soluble Reactive Phosphorus (SRP), Particulate Organic P, *Prochlorococcus*/ *Synechococcus*/ Picoeukaryote/ Nanoeukaryote cell counts

These data may be updated as additional samples or stations are processed by the principal investigators of each dataset. Another 48 metadata variables not listed here were collected aboard the GO-SHIP, PML AMT, and NSF cruises and may be available upon request from CCDHO, BODC, or SOCCOM.

*C13.5 is a partial occupation of the A13.5 GO-SHIP line that was aborted due to COVID-19. Thus, CTD casts corresponding to DNA collection were only performed at 8 stations.

Due to their rapid generation times and high diversity, microbial genomes integrate the impact of environmental change^13^ and can be used as a ‘biosensor’ of subtle biogeochemical regimes that cannot be identified from physical parameters alone^12,14–16^. Thus, the fields of microbial ecology and oceanography would benefit from coordinated, high resolution measurements of marine ‘omics products (i.e., metagenomes, metatranscriptomes, metaproteomes, etc.). This dataset provides an important example of the benefits of a high spatial and temporal resolution sampling regime. In addition, our data highlights the need for increased sampling of marine metagenomes in the Central and Western Pacific Ocean (Fig. 1), areas above 50°N and 50°S (Fig. 2), and below the euphotic zone. We hope and expect that these challenges will be addressed by the scientific community in the coming decade.

## Methods

On all cruises, whole (i.e., no size fractionation) surface water was collected via either the Niskin rosette system (depth ~3–5 m) or the ship’s circulating seawater system (depth ~7 m). Between 2–10 L of surface water (Table 1) was collected in triple-rinsed containers and gently filtered through a 0.22 μm pore size Sterivex filter (Millipore, Darmstadt, Germany) using sterilized tubing and a Masterflex peristaltic pump (Cole-Parmer, Vernon Hills, IL). DNA was preserved with 1620 μL of lysis buffer (4 mM NaCl, 750 μM sucrose, 50 mM Tris-HCl, 20 mM EDTA) and stored at −20 °C before extraction.

To extract DNA (modified from Bostrom *et al*. 2004)^17^ Sterivex filters were incubated with 180 μL lysozyme (3.5 nM) at 37 °C for 30 minutes followed by an overnight 55 °C incubation with 180 μL Proteinase K (0.35 nM) and 100 μL 10% SDS buffer. DNA was extracted from the Sterivex with 1000 μl TE buffer (10 mM Tris-HCl, 1 mM EDTA), precipitated in an ice-cold solution of 500 μL isopropanol (100%) and 1980 μL sodium acetate (3 mM, pH 5.2), pelleted via centrifuge for 30 mins at 4 °C, and resuspended in TE buffer in a 37°C water bath for 30 min. Next, DNA was purified using a genomic DNA Clean and Concentrator kit (Zymo Research Corp., Irvine, CA). Finally, DNA concentrations were quantified using a Qubit dsDNA HS Assay kit and Qubit fluorometer (ThermoFisher, Waltham, MA).

A total of 971 metagenomic libraries from 932 locations were prepared using Illumina-specific Nextera DNA transposase adapters and a Tagment DNA Enzyme and Buffer Kit (Illumina, San Diego, CA, cat. no. 20034197) (modified from Baym *et al*. 2015)^18–20^. Nextera adapter sequences to be used for bioinformatic quality trimming are: 5′-TCG TCG GCA GCG TCA GAT GTG TAT AAG AGA CAG-3′ and 5′-GTC TCG TGG GCT CGG AGA TGT GTA TAA GAG ACA G-3′. Custom Nextera DNA-style 8 bp unique dual index (UDI) barcodes I7 (5′-CAA GCA GAA GAC GGC ATA CGA GAT [NNN NNN NN]G TCT CGT GGG CTC GG-3′) and I5 (5′-AAT GAT ACG GCG ACC ACC GAG ATC TAC AC[N NNN NNN N]TC GTC GGC AGC GTC-3′) were used to multiplex the metagenomic libraries. A total of 1 μL of 2 ng μL^−1^ DNA was added to 1.5 μL tagmentation reactions (1.25 μL TD buffer, 0.25 μL TDE1) and incubated at 55 °C for 10 minutes. After tagmentation, product (2.5 μL) was immediately added to 22 μL reactions (1.02 μM per UDI barcode, 204 μM dNTPs, 0.0204 U Phusion High Fidelity DNA polymerase and 1.02X Phusion HF Buffer [ThermoFisher, Waltham, MA] final concentration). Barcodes were annealed to tagmented products using the following polymerase chain reaction (PCR): 72 °C for 2 min., 98 °C for 30 s., followed by 13 cycles of 98 °C 10 s., 63 °C 30 s., 72 °C 30 s., and a final extension step of 72 °C for 5 min.

To quality control tagmentation products, dimers that were less than 150 nucleotides long were removed using a buffered solution (1 M NaCl, 1 mM EDTA, 10 mM Tris-HCl, 44.4 M PEG-8000, 0.055% Tween-20 final concentration) of Sera-mag SpeedBeads (ThermoFisher, Waltham, MA). Metagenomic libraries were quantified using a Qubit dsDNA HS Assay kit (ThermoFisher, Waltham, MA) and a Synergy 2 Microplate Reader (BioTek, Winooski, VT). Libraries were then pooled at equimolar concentrations. Pooled library concentration was verified using a KAPA qPCR platform (Roche, Basel, Switzerland). Finally, dimer removal as well as read size distribution were checked using a 2100 Bioanalyzer high sensitivity DNA trace (Agilent, Santa Clara, CA).

54 samples were sequenced on two Illumina HiSeq 4000 lanes using 150 bp paired-end chemistry with 300 cycles (Illumina, San Diego, CA). A total of 666 samples were sequenced on three Illumina NovaSeq S4 flowcells and an additional 251 samples were sequenced on a combination of S1 and SP flowcells using 150 bp paired-end chemistry with 300 cycles. The sequencing strategy produced a total of 2.42 × 10^10^ reads, or 3.65 × 10^12^ bp. The median number of bases per sample was 3.41 billion (range: 61,400,000–21.4 billion). Prior to read trimming and quality filtering, 74% of all forward and reverse reads had an average quality score ≥Q25 (Table 1). The sequencing cost per bp in US dollars was $8.2 × 10^−9^.

## Data Records

The majority of the samples here were collected under the auspices of the international GO-SHIP program and the national programs that contribute to it^21–24^. Links to publicly available metadata variables collected via CTD cast are provided in Table 2. A comprehensive data directory of all metadata resources, including those that were collected and may be requested from individual PIs, is available through GO-SHIP and the Carbon and Climate Hydrographic Data Office (CCDHO) under a Public Domain Mark (PDM).

Metadata variables from the AMT-28 cruise are hosted by the British Oceanographic Data Centre (BODC)^25^ and the Southern Ocean Carbon and Climate Observations and Modeling project (SOCCOM).

The BVAL46, AE1319, and NH1418 cruises were collected as a part of the “Biological Controls on the Ocean C:N:P Ratios” project funded by the NSF Division of Ocean Sciences^26–29^. Data associated with these deployments are hosted by the NSF Biological and Chemical Oceanography Data Management Office (BCO-DMO) under Project 2178 and are archived by the Woods Hole Open Access Server (WHOAS) under a Creative Commons BY 4.0 (CC BY 4.0) license.

All sequencing products associated with the Bio-GO-SHIP program can be found under BioProject ID PRJNA656268 hosted by the National Center for Biotechnology Information Sequence Read Archive (SRA)^30^. SRA accession numbers associated with each metagenome file are provided in Supplementary Table 1.

## Technical Validation

To ensure that no contamination of metagenomes occurred, negative controls were used. To ensure optimum paired-end short read sequencing, a 2100 Bioanalyzer high sensitivity DNA trace (Agilent, Santa Clara, CA) was used for each library to confirm that ~90% of the sequence fragments were above 200 bp and below 600 bp in length (Table 3). A Qubit (ThermoFisher, Waltham, MA) and a KAPA qPCR platform (Roche, Basel, Switzerland) were used to ensure that all pooled libraries were submitted for sequencing at a concentration > 15 nM.

**Table 3:** Sequencing run breakdown of Bio-GO-SHIP metagenomes including technical validation statistics.

Run	Cruises	Illumina Platform	Sample Count	Library Concentration (nM)	Fragments 200–600 bp
1	I09N	HiSeq	24	4.1*	84.02%*
2	I09N, AE1319, BVAL46, NH1418	HiSeq	30	16.52	98.85%
3	I09N	NovaSeq	215	16.29	86.34%
4	P18, AMT-28, AE1319, BVAL46, NH1418	NovaSeq	203	35.64	93.87%
5	I07N	NovaSeq	248	32.25	91.72%
6	C13.5	NovaSeq	251	23.15	87.51%

*Run 1 was concentrated via SpeedVac to 15 nM and bead size-selected such that 90% of fragments were between 200–600 bp by the UC Davis Genome Center DNA Technologies Core prior to sequencing. Final values for this run are not available.

## Usage Notes

The genomic data described here have not been pre-screened or processed in any way. We recommend quality control parameters. Prior to our sequence analysis in subsequent projects, we removed adapter sequences, performed sequence quality control, and ensured there was no contamination from common genomic add-ins such as Phi-X using the following code parameters:

Trimmomatic (v0.35): PE ILLUMINACLIP:NexteraPE-PE.fa:2:30:10 SLIDINGWINDOW:4:15 MINLEN:36

BBMap (v37.50): bbduk.sh -Xmx1g ref = /BBMap/37.50/resources/phix174_ill.ref.fa.gz k = 31 hdist = 1

Nutrient data (NO_3_, NO_2_, PO_4_, SiO_4_) collected by SOCCOM and funded by the National Science Foundation are available from the AMT-28 transect through the CCHDO (http://cchdo.ucsd.edu, search on SOCCOM).

## Supplementary information

Supplementary Table 1

## Data Availability

Custom scripts were not used to generate or process this dataset. Software versions and non-default parameters used have been appropriately specified where required.
